# The Efficacy and The Safety of Ultrasound-guided Ablation Therapy for Treating Papillary Thyroid Microcarcinoma

**DOI:** 10.7150/jca.36289

**Published:** 2019-08-28

**Authors:** Jifan Chen, Jing Cao, Fuqiang Qiu, Pintong Huang

**Affiliations:** Department of Ultrasound, The Second Affiliated Hospital, Zhejiang University School of Medicine, Hangzhou, Zhejiang, China.

**Keywords:** papillary thyroid microcarcinoma, ultrasound-guided ablation, thermal ablation, laser ablation, radio-frequency ablation, microwave ablation

## Abstract

The prevalence of papillary thyroid microcarcinoma (PTMC) increases rapidly all around the world, but the management of PTMC hasn't reached a consensus. Recently, ultrasound-guided (US-guided) ablation therapy was introduced as a feasible treatment for low-risk PTMC. The clinical application of US-guided ablation therapy needs doctors' effort to investigate the efficacy and the safety of US-guided ablation in treating PTMC carefully. Although the present evidence showed some limitations, such as short-term study time spans and no randomized control design, in our perspective, US-guided thermal ablation therapy has good short-term efficacy and safety and is a promising PTMC's treatment in future clinical practice.

## Introduction

Papillary thyroid microcarcinoma (PTMC), a frequent type of papillary thyroid carcinoma (PTC), whose longest diameter is <10 mm defined by World Health Organization (WHO), is increasing rapidly during recent decades all around the world 1. Due to the development of resolution of ultrasound device and relevant technologies, such as ultrasound elasticity imaging, fine-needle aspiration (FNA) biopsy and core-needle aspiration (CNA) biopsy, more and more patients were diagnosed with incidental PTC in which PTMC accounts for 87% 2. Although incidence rate of PTMC increased, the prognosis of PTMC was remarkably excellent because of its indolent feature based on a review from Brito, J. P. et al. who reported that patients with small localized PTC have a 99% survival rate after 20 years 3. The prevalence of PTMC was a significant burden worldwidely, not only threating patients' deposits but also doing harm to patients' psychological health by causing unnecessary anxiety.

Since then, there has been controversy amongst clinicians about treating this disease in an aggressive or a conservative way. The treatments of PTMC varied among experts of thyroid cancer. The 2015 America thyroid Association (ATA) guideline for thyroid cancer recommended active surveillance (AS) in patients with incidental PTMC 4. On the other hand, 2016 Chinese expert consensus and guidelines recommended surgical procedure as the first-line treatment for this disease 5. What's more, other experts recommended treatments like total thyroidectomy/lobectomy with or without prophylactic central neck dissection and suppression of serum thyroid-stimulating hormone with levothyroxine 2. Diverse treatments of PTMC have both advantages and disadvantages and should be applied to suitable patients based on carefully clinical risk stratification. Considering the indolent feature of PTMC, treatments' efficacy, safety and even medical economy should be taken into consideration. As to surgical procedure, high patients' costs and long hospital stay were unavoidable and, in some case, patients' post-operative life quality will be even worse than pre-operation if related complication happens 6. As to AS, some patients' progressing anxiety and heavily psychological burden, because of the tumor diagnosis and the probability of progression, could lead to decreasing life quality even if doctor had interpreted the benign-like feature of this disease.

In China, Korea, Italy and Japan, some clinicians regarded US-guided ablation procedures as acceptable treatments for patients with low-risk PTMC 7-10. These image-guided minimally invasive technologies (e.g., ethanol ablation (EA), microwave ablation (MWA), laser ablation (LA), radiofrequency ablation (RFA) and high-intensity focused ultrasound (HIFU)) were introduced in thyroid disease for treating benign nodules, local recurrences of thyroid tumors and other disorders in order to get an ideal efficacy and reduce adverse events 11, 12. Although US-guided ablation therapy was effective in benign nodules and local recurrences of thyroid tumors, its efficacy and safety in treating PTMC remain unknown and arise controversies. A few years ago, Papini et al.^10^ firstly used LA for treating incidental PTMC in one patient who couldn't tolerate surgical operation well and reported a perfect outcome after 24 months follow-up. Following with Papini's work, the US-guided ablation technologies, especially thermal ablation technologies, started to be applied to low-risk PTMC, but published researches in this field were still rare and required to be encouraged. This review aims to summarize US-guided ablation technologies which are representatively applied to treat PTMC, discuss the efficacy and the safety of these US-guided ablation procedures and seek potential direction for further studies focusing on this topic.

## Search Strategy and Eligible Criteria of Reference

A comprehensive literature search of PubMed, PubMed Central (PMC), EMBASE and Cochrane Library (CENTRAL) was done to find suitable articles up to July, 2019. The search terms were based on combination Medical Subject Heading terms and free words (synonym), and restricted language in English. The search terms included (“Papillary Thyroid Microcarcinoma” OR “PTMC”) AND (“Ablation techniques” OR “Ablation” OR “Ultrasound therapy” OR “High-intensity Focused Ultrasound” OR “USG-HIFU” OR “Radiofrequency ablation” OR “PLA” OR “percutaneous laser ablation” OR “laser ablation” OR “LA” OR “microwave ablation” OR “microwave”).

**Figure 1:**
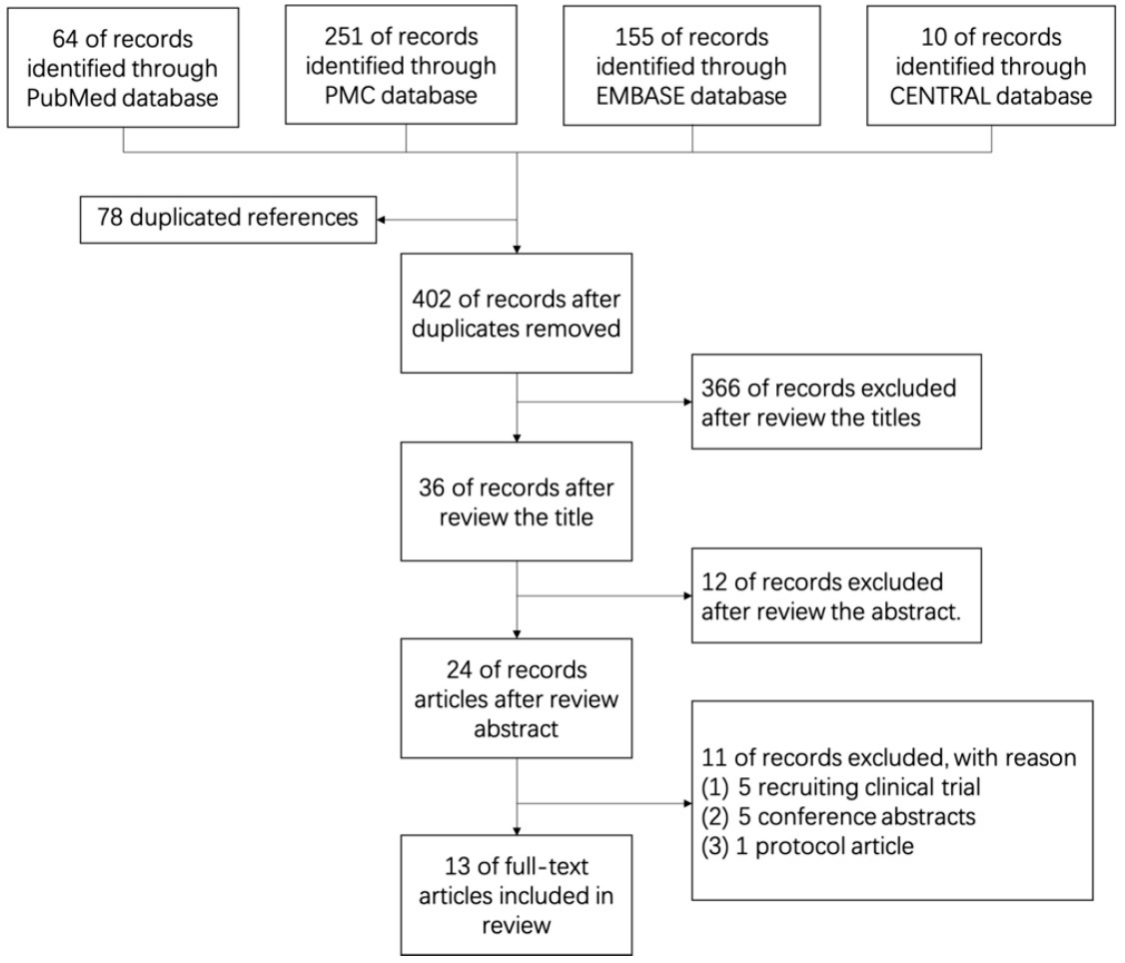
Flow Graph of Review

After searching medical databases such as PubMed, PMC, EMBASE and Cochrane Library (CENTRAL) carefully, 480 English references in which 64 from PubMed, 251 from PMC, 155 from EMBASE and 10 from CENTRAL were found before July, 2019. Of all 480 references, 78 duplicated references were removed, 378 references were removed by 2 independent reviewers (FQQ and JC) because of low relevant after reading titles (366) and abstracts (12) elaborately. Further, 5 recruiting clinical trials were removed, 5 conference abstracts were removed and 1 research protocol article was removed after reading the full-text. Finally, 13 original articles were enrolled in this review (Figure 1, Table 1). All cited studies have gotten informed consent from each study participant and protocol approval by an ethics committee or institutional review board.

## Technologies applied in the clinical practice of PTMC

In the last few years, some medical doctors tended to apply thermal ablation technologies to treat PTMC. These substituted procedures were performed with early clinical stage of tumor in patients with PTMC. Most of the clinical researchers enrolled T1aN0M0 PTMC patients who were diagnosed by imaging technologies, such as conventional ultrasound, contrast-enhanced ultrasound (CEUS), computed tomography (CT) and magnetic resonance imaging (MRI) 9, 13.

**Figure 2:**
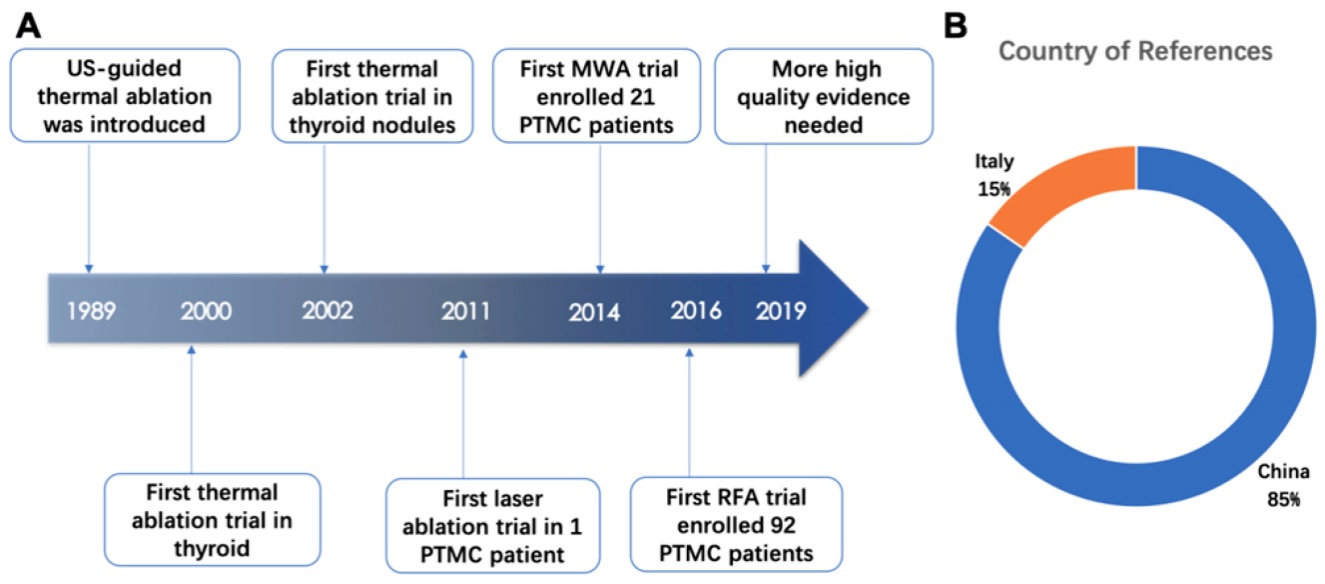
Application of US-guided thermal ablation in the thyroid

US-guided thermal ablated technologies were introduced into clinical practice since 1989 14-16 and provided ideally hyperthermal injury in target cell whose microenvironment drastic changed and cell membrane even subcellular structure damaged 14. In 2000, the first trial of thermal ablation in thyroid tissue was performed and doctors have extended US-guided thermal ablation for management of thyroid nodules since 2002. As a new field, until 2011, clinical researchers started applying US-guide thermal ablation technologies in PTMC (Figure 2A).

**Figure 3:**
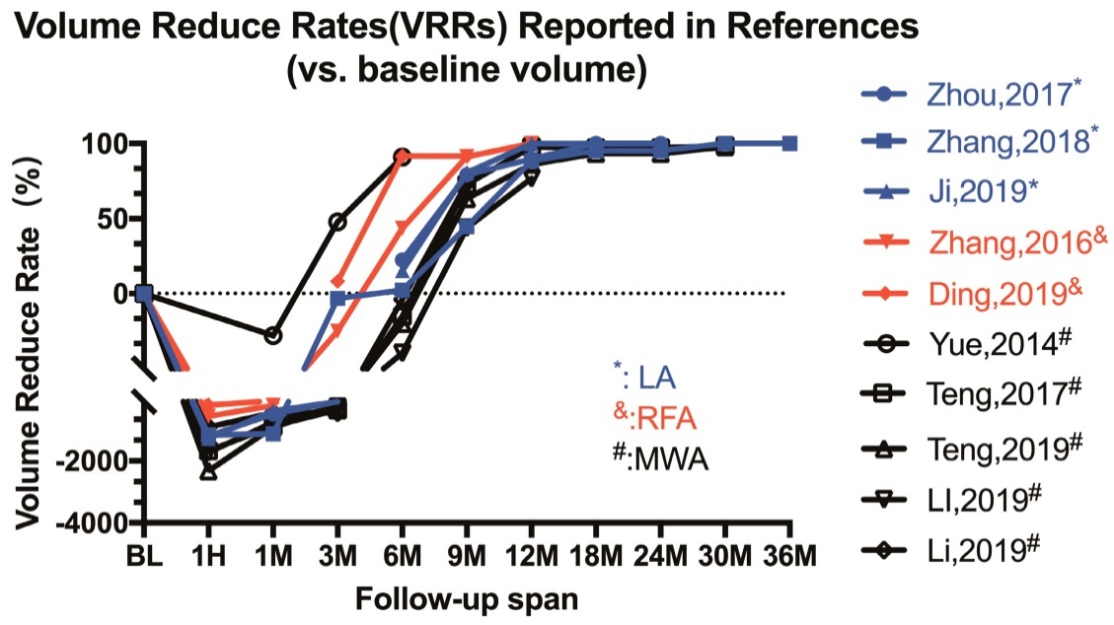
Line Chart of Volume Reduce Rates (VRRs) Reported in References.

Among the thirteen articles included in this review, eleven articles came from China and two came from Italy (Figure 2B). The efficacy of the US-guided thermal procedures in patients with PTMC could be demonstrated by post-operative CEUS, post-operative pathological exanimation and long-term follow-up 17. Almost all references prefer to report volume reduction rate (VRR) as an index of efficacy of ablation therapy. The VRRs were reported in ten articles and displayed in Figure 3 and Table 2. Besides, the safety of ablation therapy could be demonstrated by operatively relevant complications and the result displayed in Table 3.

## US-guided Laser Ablation (LA)

The laser technology had broad clinical application in many diseases, such as unresectable breast cancer 18, renal tumor 19, hepatocellular carcinoma (HCC) 20, thyroid nodule 21, 22 and, more advanced, photodynamic cancer therapy 23, 24. Since US-guided LA was applied in PTMC firstly by Papini et al. 10, four more articles were written about LA 9, 17, 25, 26. Zhang et al. 17 enrolled 64 PTMC patients in their study and demonstrated that the original volume of PTMC increased from 41.0 ± 40.4 mm^3^ to 517.6 ± 262.94 mm^3^ after ablation and decreased to 1.8mm ± 6.7 mm^3^ after 25.7 months mean follow-up. The lesion of patients with 36 months follow-up completely disappeared (VRRs=100%). Likewise, Valacvi et al. 25 performed the pathological and histological results demonstrated that the cell morphology became distortion or shrinkage and chromatin became condensation when cell was heated from laser beam. These results supported LA's good effectiveness in treating PTMC. However, some studies reported residue after LA ablation, which could be detected by post-ablation CEUS. Zhou et al. reported 3.3% (1/30) residual rate and Zhang et al. announced 3.12% (2/64) residual rate 9, 17. In study of Ji et al. 8 incomplete ablation lesions were found after the first LA ablation 26, in that case, a secondary ablation was applied. The LA procedure accompany with CEUS was recommended basing on above studies. Besides, the LA shows perfect post-operative safety, as we could see that almost no serious adverse events after LA procedure among 5 relevant studies. (1 patient with pain received dezocine injection and 1 patient with serum hormone abnormalities) (Table 3) The reasons for little post-operative complication might interpret by LA's minimally invasive and precise feature using a 21-gauge needle and well experienced radiologists who performed the procedure. Although parts of the reasons for excellent efficacy might be too short follow-up time and insufficiency of patient's number, the US-guided LA still could be a rational treatment for selective PTMC patients. Unfortunately, the all LA articles were retrospective design and performed without a comparable group, the efficacy and the safety in clinical practice still need more high-quality evidence to prove.

## The procedure of US-guided LA

During LA procedure, patients who keep supine positions had local anesthesia by 1% lidocaine. The radiologist, assistant and trained nurse work with seriously aseptic criteria. The LA was commonly performed with a continuous-wave neodymium yttrium-aluminium-garnet (Nd: YAG) laser source operating at 1064 nm and an optical beam-splitting device. Optical fibre transmits light to tissue and the heat of tissue rises with conventional or contrast ultrasound guidance. Under the laser beam, the tissue vaporized, coagulated without bleeding and finally heal with fibrosis 27. The ablated region should be extended tumor edge to prevent marginal residue and recurrence.

## US-guided Radiofrequency Ablation (RFA)

US-guided RFA were introduced to treat various cancer such as pancreatic tumor 28, thyroid tumor 29, 30 and primary or secondary liver cancer 31, 32 and so on. Two original articles focusing on US-guided RFA in patients with PTMC was published. Zhang et al. 33 enrolled 92 patients with 98 PTMCs and demonstrated that PTMC volume increased from 112.7 ± 105.8 mm^3^ to 749.8 ± 105.8 mm^3^ after ablation and decreased to 9.9±19 mm^3^ after 7.8±2.0 months mean follow-up. Three months after RFA, the author performed the US-guided core-needle biopsy (CNB) to evaluate the efficacy of RFA in the ablated region and reported no residual lesion. Ding et al. 34 reported excellent efficacy of RFA in treating PTMC, almost 97.4% complete absorbed lesions were reported. But in Zhang's study, the rate of complete absorption is 33.5%. Within the follow-up span, no patients suffered from PTMC's recurrence. Besides, the procedure of RFA, as reported in Zhang's and Ding's paper, was generally safe and only few minor complications could happen. After RFA procedure, one patient felt moderate pain and received pain-killer for this symptom, four patients had transient hoarseness which recovered in three hours and no patient suffered dysphagia, hoarseness, permanent hypoparathyroidism, hematoma, transient or permanent hypothyroidism 33 (Table 3). By the way, 2 RFA references both enrolled a few patients with more than one lesion and might extend usage of thermal ablation therapy which was usually performed in unifocal lesion. References above reported a good result using RFA to treat PTMC but with a low evidence level in evidence-based medicine (EBM), further studies still need to be encouraged to evaluate the efficacy and the safety of RFA in PTMC field.

## The procedure of US-guided RFA

Patients were in supine positions and had local anesthesia by 1% lidocaine. The procedure complied with the aseptic criteria seriously. If the distance between the tumor and critical cervical structures was less than 5 mm, normal saline was injected to form at least 1 cm distance between the tumor and the critical structure to prevent the unwilling thermal injury. The RFA procedure was performed using moving-shot technique and output power was 3-5W with a bipolar RFA generator and an 18-gauge bipolar PF applicator with a 0.9 cm active tip 33. The agitation of the ions surrounding the electrode results in frictional heat and subsequent tissue was necrotic 35. The RFA extent exceeded the tumor edge to prevent marginal residue and recurrence and target ablation area had changed to hyperechoic zone after the procedure.

## US-guided Microwave Ablation (MWA)

MWA was introduced as a treatment of various disorders and disease, for example, endometrial ablation 36, 37, liver carcinoma 38-40, benign thyroid nodule 41 and so on. Six articles including three single-arm clinical trials and three comparable clinical trial reported by Chinese authors focusing on MWA therapy in PTMC field. Li's work had the longest follow-up time in thermal ablation field, in which patients received at least 42 months follow-up. Lesions' longest diameter reduced from 4.29±1.37mm pre-operatively to 1.67±1.12mm post-operatively 42. The VRRs of PTMC at time point of final follow-up were reported 91%, 98.7%, 97.8%, 89.6% and 73.8% respectively among articles 13, 42-45. (Table 2) Teng reported 95.2% (20/21) and 84.5% (174/206) completely absorbed rate after MWA procedure, but Yue and Li only reported 19% (4/21) ,15.2% (7/46), 20.24% (34/168) (Table 3) 13, 43. The differences among studies could be explained by inconsistently therapeutic parameters (Table 4), varied patients' baseline features and experience of procedure among radiologists. In addition, no residue was identified among six MWA studies. Three articles investigated MWA procedure comparing with surgery and reported that MWA, as a minimally invasive treatment of PTMC, embraced lower incidence rate of complications and without decreasing the therapeutic effect 6, 42, 45. The hospital stay, surgery/procedure time is significantly shorter in MWA group than surgery group (Table 5). Although 7 patients in MWA group were detected as recurrence during follow-up in Li's study, no statistically significant was found comparing with the surgery group 45. Basing on the comparable studies, surgery causes more complications than MWA procedure (Table 5) and the *P* value is lower than 0.05 in all three studies. No direct evidence was reported by researchers if we want to compare the complications among different thermal ablation technologies (LA, RFA and MWA). According to data we collected in Table 3, it seems that more minor complications, including transient hoarseness, burning sensation, choking and coughing, toothache, voice slightly change, region discomfort and neck swelling, were reported during or after MWA procedure than LA or RFA procedure. This difference among thermal technologies could be partly interpreted by MWA's better efficiency to achieve higher heat and a 16-17 gauge needle in MWA procedure which causes larger ablated region 14.

Still, more high-quality evidence, especially randomized controlled trial (RCT) with blind design, is necessary and should be encouraged in low-risk PTMC.

## The procedure of US-guided MWA

Patients lied down with supine position and neck exposure. Under the ultrasonic guidance, ablation needle was inserted into the nodules' lower pole and move to its upper pole. The procedure was performed by an experienced radiologist using 16 or 17-gauge needles with different MWA systems reported in two papers^42^, ^43^ (KY2000, Kangyou Medical Instrument, Nanjing, China, ECO-100A1; YIGAO Microwave System Engineering Co.Ltd, Nanjing, Jiangsu Province, China). The output power was varied among papers, which ranged from 20-40W and therapeutic procedure was sustained until the ultrasound imaging of the nodule became hyperechoic. All procedure was performed compiling with seriously aseptic criteria. The heat generated by MWA system broke cell membrane and cause coagulation necrosis in tumor tissue.

## Other Technologies

Ethanol ablation (EA) in thyroid lesions had been widely investigated many years ago, especially in cystic or predominantly cystic lesions 46-48. Sung et al. reported that EA could be the first-line treatment in cystic thyroid nodules and therapy of EA was less expensive than RFA while having the same efficacy 47. But the solid type of thyroid cancer which accounts for 82-91% proportion in all thyroid cancer limited EA's usage in this field 49, 50. In Henrichsen and his colleagues' report, 88% thyroid cancer were predominant solid, 9% were <50% cystic, and only 3% were more than 50% cystic 51. Although, Monchik and his collogues tried to combine RFA and EA to treat local and distant recurrence in well-differentiated thyroid carcinoma 52, whether treatment combining RFA and EA could be applied to PTMC patients and whether combination therapy could provide better effect than RFA or EA alone was still unknown.

A new technology, High-intensity focused ultrasound (HIFU), was applied in thyroid disease recently 53. Esnault et al. performed HIFU in eight ewes to destroy a defined area of thyroid and reported satisfied necrotic zone with peripheral fibroblastic granulation tissue 54. Then, Esnault et al. further reported the first HIFU study in human thyroid nodules and showed HIFU's potential efficacy by histologic and ultrasonic examination 53. Lang B.H. et al. applied HIFU into benign thyroid diseases such as Gravels Disease 55 and large-size benign thyroid nodules 56, HIFU had excellent safety and efficacy in treating thyroid disorders above. However, in PTMC field, the HIFU research was not published yet and needed researcher to investigate step by step.

## Discussion

Some scientists started focusing on US-guided thermal technologies to treat PTMC since 2011 10. But, US-guided thermal technologies weren't mentioned as a therapy of PTMC and were reported only as a feasible local treatment for recurrent metastases in the 2015 ATA guideline 4. As a result of lacking the high-quality evidence and long follow-up span studies to potently prove efficacy and safety of US-guided ablation technologies, it's not surprised that US-guided ablation therapy didn't become a widely acceptable method for PTMC's management. But in our opinions, it's still a promising method.

In our review, we summed up the published articles which target on PTMC field to prove the efficacy and the safety of US-guided ablation technologies. The results extracted from these clinical trials were exciting to clinical practicer, nevertheless the results based on low-quality evidence in EBM field. Among thirteen papers, the post-operative VRRs and total absorb rates ranged from 73.8% to 100% and 15.2% to 97.4% respectively with few serious complications after ablation procedures.

Of three US-guided technologies, LA seems to encounter the fewest complications. However, LA might have a more incomplete ablation rate (1/30, 2/64) than the other ablation technologies. The phenomenon could be explained that the ablated region of MWA was the largest, RFA ranked secondly, LA was minimal, as we found that the needle size was reduced respectively. Some experts might worry about the recurrence which might appear in or near the scar, especially in LA procedures. Firstly, the residue and recurrence in studies were quite rare, which is accordant with our clinical observation in our hospital. Secondly, in our opinion, the CEUS have good diagnostic performance in detecting the residue after ablation and recurrence of PTMC with regular follow-up strategy. If there is any sign of enhancement in ablated region, the accuracy biopsy with CEUS guidance and the second ablation procedure could also perform. Thirdly, the recurrence in scar area seems less persuadable as the scar region is totally necrotic by adequate heat generated by thermal ablation system.

Although US-guided ablation could provide excellent effect and few post-operative complications, as a rational clinical performer, it is necessary to choose suitable PTMC patients to accept US-guided ablation procedure. Majority of researchers used US-guided ablation for patients with low-risk PTMC 13 or patients who can't stand surgery 10. Low-risk PTMC was defined as the largest diameter <10mm PTC without clinical-invisible cervical and distant metastasis (TNM stage: T1aN0M0). Actually, low-risk PTMC may not have truly low risk due to the insensitivity for detecting clinical suspicious lymph node by existing imaging technologies. In Joo's 57 study, almost 8.6%-26.3% central lymph node was found in surgery which was undetected by pre-surgery image diagnosis. Although pre-operative evaluation could underestimate the risk of PTMC, the pre-operative diagnosis could also provide parts of clues about essential progressing factors. BRAF V600E mutation was reported as a crucial prognosis factor in PTC according to many kinds of researches and could be detected by fine-needle aspiration (FNA) biopsy 58, 59. In PTMC, Chen Y et al. reported a meta-analysis, including 2247 PTMC patients, which revealed that BRAF(V600E) mutation patients were associated with recurrence and BRAF(V600E) might be helpful in stratifying PTMC patients before the operation 60. Besides, some demographic and ultrasonic features might potently imply malignance of PTMC, such as age >45, male sex, tumor size, multifocality, extrathyroidal extension 61, 62. However, some potently prognostic factors could only be obtained by surgery. The subtype of PTMC was investigated by some researchers to differentiate risks in different subtypes. As Liu et al^63^ reported, the encapsulated follicular variant of papillary thyroid carcinoma (EFVPTC), a subtype of PTMC, doesn't have the signs like lymph node metastases or recurrence. To the contrary, the tall cell variant of PTMC is associated with aggressive features and more likely to advance 64. In our perspective, before preforming US-guided ablation procedures, the PTMC patients should be separated by pathological, clinical and image features to get a rational risk stratification. However, it might cause some bias and inaccuracy regarding to the study result's extrapolation if investigators only take ultrasonic features which is conveniently acquired to identify low-risk PTMC.

Nowadays, comparing with aggressive surgery, many researchers noted that active surveillance (AS), which initiated in Kuma Hospital and Cancer Institute Hospital in the mid-1990s, was recommended as the first-line treatment for low-risk PTMC 65, 66. The first study about AS was published in 2003, which is earlier than US-guided ablation therapy, and its follow-up span lasts more than eight years 67, which indicated that clinical recognition of AS was even much earlier than US-guided ablation therapy. As time goes by, we positively think that US-guided ablation therapy could earn a place in PTMC's treatment as more high-quality evidence and longer follow-up span studies showing up.

## Existing Limitations

Some limitations in present studies were displayed below. (1) The evidence level of published references about PTMC was not high in EBM. (2) Because of the different baseline data of enrolled patients, the comparison among studies was inaccurate. (3) Parameters of the US-guided ablation procedure haven't reached consistent among different studies, even if studies focused on the same thermal ablation technology. (4) Comparing with AS studies, short follow-up time span restricted efficiency of US-guided ablated studies' result. (5) The clinical risk stratification of PTMC remains inaccurate due to insufficient prognostic factors which could be detected pre-surgery. (6) The review only included studies writing in English.

## Further Directions

In US-guided ablation therapy to treat PTMC field, several single-arm and comparable clinical trials with relative short follow-up time had been published, the more high-quality evidence such as randomized control trial (RCT), prospective cohort study or case-control study with a long-time follow-up span were needed for further investigation. Here are some questions which need to be solved in the future. Whether US-guided ablation procedures could be applied in patients regardless of subtypes of PTMC when lesions were still small or in an early stage. Besides, whether US-guided ablation therapy could extend its application for other early-stage pathological subtypes of thyroid cancer. Furthermore, researchers who tended to compare US-guided ablation with surgery might cause bias in patients' selection and data collection because of the retrospective feature. Also, the comparison between AS and US-guided ablated therapy, introduced as alternative treatments of surgery in PTMC, has important clinical significance and nearly no studies focused on this topic yet. So, the RCT in PTMC field should be designed to solve these problems. Finally, the indirect comparison between different thermal technologies was published in thyroid nodules in a meta-analysis using Bayesian network meta-analysis 68, but indirect/direct comparison among different technologies, in PTMC field, were not published yet.

## Conclusions

This review displays present usage of US-guided ablation therapy in PTMC and explains the efficacy and the safety of US-guided ablation therapy in treating the lesions. This review suggests some further research directions which could complement powerful evidence. Although the present studies showed some limitations and might insufficient support the clinical application of the minimal-invasive therapy, US-guided ablation therapy are still a promising treatment and have broad clinical prospects for the management of low-risk PTMC.

## Figures and Tables

**Table 1:** Characterization of Included Studies in this Review.

Number	Method	Author	Year	Country	Institution	Study Design^ &^	Center	No. of Patients/Nodules#
1	LA	Zhou	2017	China	Rui Jin Hospital	R	1	30/30
2	LA	Zhang	2018	China	Rui Jin Hospital	R	1	64/64
3	LA	Valcavi	2013	Italy	Arcispedale Santa Maria Nuova and Clinical Cancer Research Institute (IRCCS)	N	1	3/6
4	LA	Papini	2011	Italy	Regina Apostolorum Hospital	N	1	1/1
5	LA	Ji	2019	China	The Affiliated Suzhou Hospital of Nanjing Medical University	R	1	37/37
6	RFA	Zhang	2016	China	General Hospital of Chinese PLA	P	1	92/98
7	RFA	Ding	2019	China	Renji Hospital	R	1	37/38
8	MWA	Yue	2014	China	Yantai Affiliated Hospital, Binzhou Medical University	P	1	21/21
9	MWA	Teng	2017	China	China-Japan Union Hospital of Jilin University	N	1	15/21
10	MWA	Teng	2019	China	China-Japan Union Hospital of Jilin University	R	1	185/206
11	MWA	Li	2018	China	Beijing Friendship Hospital	R	1	46/46
12	MWA	Xu	2018	China	Fudan University Affiliated Shanghai Fifth People's Hospital	R	1	41/-
13	MWA	LI	2019	China	Beijing Friendship Hospital	R	1	168/-

&: R=Retrospective Study P=Prospective Study; -: not available or missing value.

**Table 2:** Efficacy of ablation in volume reduction at different time point.

No	Method	Author	Year	Pre-ablation diameter	Pre-ablation volume	Post-ablation volume	Post-ablation Follow-up (mm^3^ or %)								
							1M	3M	6M	12M	18M	24M	30M	36M	42M
1	LA	Zhou	2017	4.8±1.2	43.7±37.8	566.8±267.7	259.8±149.6	87.1±69.2	33.8±24.3	9.1±13.5	4.5±5.7	0	-	-	-
2	LA	Zhang	2018	4.6±1.5	41.0±40.4	517.6±262.9	-11.30%	-3.30%	2.20%	44.70%	88.50%	95.30%	96.80%	100.00%	100.00%
3	LA	Ji	2019	5.1±3.4	52.8±30.6	726.5±187.2	258.1±97.6	102.7±39.5	44.7±24.3	10.2±8.7	5.7±6.5	2.1±1.3	-	-	-
4	RFA	Zhang	2016	5.8±2.2	112.7±105.8	749.8±594.4	355.2±362.7	140.8±215.1	63.4±171.6	9.9±19	0	-	-	-	-
5	RFA	Ding	2019	6.77±1.92	120±100	370±260	200±210	110±210	10±40	10±30	-	-	-	-	-
6	MWA	Yue	2014	7.3±3	89.5±20.1	-	-28%	48%	91%	-	-	-	-	-	-
7	MWA	Teng	2017	5.8±2.5	174.0±259.1	3099.4±3004.5	1694.8±2227.6	793.2±970	199.3±245.6	47±87.2	3.9±12.9	5±13.7	3.2±11.3	2.3±10.5	-
8	MWA	Teng	2019	5.3±1.91	100.1±92.9	2421.9±1279.0	1014.4±756.3	369.9±375.1	119.9±173.2	36.6±75.1	13.8±39.7	6.6±18.2	3.2±9.0	2.2±5.6	-
9	MWA	Li	2018	4.29±1.37	53.61±48.43	978.2±605.7	458.3±234.3	194.1±121.1	74.76±61.75	30.08±38.3	12.4±19.9	-	5.5±7.7	-	4.8±6.5
10	MWA	LI	2018	-	81.60±9.99	828.9±63.1	454.2±45.9	454.2±45.9	84.70±12.90	21.41±4.0	-	-	-	-	-

-: not available or missing value; The article will not display if they don't report the parameters in this table.

**Table 3:** Outcome of ablation procedure in Enrolled Articles.

No	Method	Author	Year	Incomplete Ablation	Recurrent	Disappear (complete absorbed)	Complication
1	LA	Zhou	2017	1	0	33%	No serious complication
2	LA	Zhang	2018	2	1	79.70%	Not mention
3	LA	Valcavi	2013	0	0	-	not mention
4	LA	Papini	2011	0	0	-	No complication.
5	LA	Ji	2019	8	1	32.40%	1 patient with pain received dezocine injection; 1 Serum hormone abnormalities;
6	RFA	Zhang	2016	0	0	33.50%	1 patient with moderate pain received painkiller; 4 transient hoarseness;
7	RFA	Ding	2019	0	0	97.40%	No complication.
8	MWA	Yue	2014	0	0	22.20%	4 transient hoarseness; burn sensation; 2 choke and cough;
9	MWA	Teng	2017	0	0	95.20%	5 burn sensation; 3 toothache; 1 transient hoarseness;
10	MWA	Teng	2019	0	1	84.50%	5 hoarseness; 11 bleeding; 21 earache or toothache; 1 another lesion in thyroid.
11	MWA	Li	2018	0	0	15.20%	2 transient hoarseness;
12	MWA	LI	2019	0	7	20.70%	6 transient voice change; 1 Persistent voice change;

-: not available or missing value; The articles will not display if they don't report the parameters in this table

**Table 4:** Parameters of Ablation Technologies in Enrolled Articles.

No	Method	Author	Year	Male/Female	Age (year)	Power	Time (s)	Needle	Follow-up (months)
1	LA	Zhou	2017	13/17	16-69	3-4W	274±57	21G	13.2 (12-24)
2	LA	Zhang	2018	23/41	42.5±12.3	3-4W	271.6±86.7	21G	25.7±8.2
3	LA	Valcavi	2013	0/3	52.3±9.3	3W	600	21G	N. A
4	LA	Papini	2011	0/1	81	3W	600	21G	24
5	LA	Ji	2019	12/25	43.9±17.6	3-4W	165.9±92.8	21G	16.5 ± 6.9
6	RFA	Zhang	2016	23/69	44.7±10.7	3-5W	450.8±230.2	18G	7.8 ± 2.9
7	RFA	Ding	2019	8/29	45.1±12.96	20W	20-120	18G	12
8	MWA	Yue	2014	6/15	52.1±13.6	40W	500±75.8	16G	11(3-22)
9	MWA	Teng	2017	6/9	48±8.8	20W	234.3±168.3	16G	36-48
10	MWA	Teng	2019	40/145	42.2±11.7	20W	N. A	16G	20.7±8.8
11	MWA	Li	2018	14/32	43.6±9.27	30W	611.4±501	17G	>42
12	MWA	Xu	2018	12/29	45.8±10.2	50W	60-300	16G	N. A
13	MWA	LI	2019	36/132	47.36±10.75	30W	20-120	17G	25.1 ± 17.3

-:not available or missing value; The article wasn't display if they don't report the parameters in this table

**Table 5:** Comparable Studies of MWA and surgery.

No	country	Author	Year	Procedure	Case	Age	Gender (M/F)	Nodules Diameter	Hospitalization (Days)	Recurrence	Surgery/Procedure Time (minutes)	Complications^*^
1	China	Li	2018									
				surgery	46	49.59±9.0	13/33	4.29±1.37	7.47±2.94^*^	0	75.80±11.23^*^	4 Dysphagia; 7 transient Hoarseness; 1 permanent Hoarseness; 1 Hematoma; 2 post-operation hyperthyroidisms; 6 transient Hypothyroidism; 9 permanent Hypothyroidism;
				MWA	46	43.63±9.27	14/32	4.49±1.55	1.3±0.51^*^	0	10.19±8.35^*^	2 transient Hoarseness
2	China	Xu	2018									
				surgery	46	46.2±11.5	16/30	8.13±1.22^*^	4.18 ± 0.55^*^	-	78.81±12.19^*^	1 pharyngeal discomfort; 2 Hoarseness; 1 Pain; 1 incision infection; 2 coughs after drinking water;
				MWA	41	45.8±10.2	12/29	8.87±1.01^*^	1.77 ± 0.71^*^	-	25.02±4.14^*^	1 Hoarseness; 1 Cough after drinking water
3	China	LI	2019									
				surgery	143	49.18±11.41	29/114	-	-	6	-	10 Transient hypoparathyroidisms; 6 Transient voice change; 1 Persistent voice change
				MWA	168	47.36±10.75	36/132	-	-	7	-	6 Transient voice change; 1 Persistent voice change

*: represent the statistically significant. Between surgery and MWA.
